# Sarcopenia in Patients With Parkinson's Disease: A Systematic Review and Meta-Analysis

**DOI:** 10.3389/fneur.2021.598035

**Published:** 2021-03-05

**Authors:** Yingying Cai, Fei Feng, Qianqian Wei, Zheng Jiang, Ruwei Ou, Huifang Shang

**Affiliations:** ^1^Laboratory of Neurodegenerative Disorders, Department of Neurology, West China Hospital, Sichuan University, Chengdu, China; ^2^Department of Geriatrics, Clinical Medical College and The First Affiliated Hospital of Chengdu Medical College, Chengdu, China

**Keywords:** sarcopenia, Parkinson's disease, systematic review, meta-analysis, prevalence, fall

## Abstract

**Background:** Parkinson's disease (PD) and sarcopenia are two common diseases in aging people. To date, the prevalence of sarcopenia in PD patients and the relationship between clinical features and sarcopenia in PD patients are not clear. The aim of the study was to (1) assess the prevalence of sarcopenia in PD patients and (2) reveal the clinical features between PD patients with and without sarcopenia.

**Method:** A systematic review was carried out through screening PubMed, EMBASE, and Cochrane database in May 2020. All study designs (case–control, cohort, and cross-sectional studies) were eligible for meta-analysis. Data of patients' characteristics, sarcopenia criteria, sarcopenia prevalence, and sarcopenia measures were retrieved. The primary outcome was estimated prevalence of sarcopenia by a pooled prevalence (%) and its 95% confidence interval (CI), using a random-effects model. The secondary outcome was the differences in clinical features between PD patients with and without sarcopenia by meta-analysis. Included articles were assessed for risk of bias. Potential sources of variation were investigated by using subgroup analyses and meta-regression.

**Result:** Ten studies were included in the review. Among them, nine were cross-sectional studies, and one was a prospective cohort study. Age of participants with PD in the studies ranged from 51.1 to 80.7 years. The estimated prevalence of sarcopenia ranged from 6 to 55.5%. The random-effects pooled prevalence was 29% (95% CIs: 0.18–0.40). When only studies at low risk of bias were considered, pooled prevalence decreased to 17% (95% CIs: 0.02–0.33), with still high heterogeneity. The incidence of falls in PD patients with sarcopenia was higher than that in PD patients without sarcopenia. There was no difference in sex ratio between PD patients with and without sarcopenia.

**Conclusion:** Sarcopenia seems to be common in patients with PD. Early assessment of sarcopenia should be implemented in PD to avoid fall and disability.

## Introduction

Parkinson's disease (PD) is a common neurodegenerative disorder that becomes increasingly prevalent with aging and results in dependency over time, despite the best treatment approaches (1). Sarcopenia was recognized as a muscle disease with low muscle mass and muscle function by WHO with a specific International Classification of Disease, Tenth Revision (ICD-10 code, M62.84) in 2016 (2). Sarcopenia is commonly seen in elderly individuals with chronic diseases, including PD (3, 4), which is an important determinant of quality of life (QoL), disability, and mortality in the elderly population (5).

Several sarcopenia definitions have been developed by different working groups or societies since the first term of sarcopenia was defined in 1988 (6) [the European Working Group on Sarcopenia in Older People (EWGSOP) (7, 8), the International Working Group on Sarcopenia (IWGS) (9), the Society on Sarcopenia, Cachexia and Wasting Disorders (SCWD) (10), the Foundation for the National Institutes of Health Biomarkers Consortium Sarcopenia Project (FNIH) (11), Baumgartner (12), Newman (13), the decreased appendicular skeletal muscle mass index (ASMMI) (14), SARC-F (a tool for screening sarcopenia risk) (15), probable sarcopenia (16), early stage sarcopenia (ESS) (17), and the Asia Working Group for Sarcopenia (AWGS) (18)]. Among which, the most used definition was developed by EWGSOP. However, the European screening algorithm of sarcopenia developed by EWGSOP has been updated to the 2nd version in 2019 (8) since the 1st version developed in 2010 (7). Until now, no worldwide consensus has yet been reached.

Sarcopenia can be assessed by the EWGSOP algorithm including the combination of a low muscle mass and a low handgrip strength (HS) or a low gait speed (GS). Muscle mass is measured by using traditional anthropometric measures—bioelectrical impedance analysis (BIA) (3, 4, 17, 19, 20), whereas several studies used more precise methods, such as dual-energy X-ray absorptiometry (DEXA) (14, 21, 22) and magnetic resonance imaging (MRI) (23). The prevalence of sarcopenia in community-dwelling older adults was reported in the wide range of 1–50% (24). The prevalence of sarcopenia in PD was higher than that of the healthy older control group matched for age and sex (3, 20, 21). However, the prevalence of sarcopenia in PD is varied among different studies. For example, some studies reported the prevalence of sarcopenia in PD with a range of 6–31.4% (3, 4, 19, 20, 22) according to the 1st version EWGSOP; other studies found the prevalence of sarcopenia as 40.4, 17.2, 58, 47.2, and 38% according to the decreased ASMMI (14), AWGS (21), SARC-F (15), probable sarcopenia (16), and ESS (17), respectively. This large discrepancy among the studies may be due to diagnostic criteria, muscle mass measurement techniques, different cut-off values for muscle mass indexes for the definition of sarcopenia, and as well as the characteristic of enrolled PD patients.

Some studies reported that sarcopenia was related to disease duration (15, 16) Hoehn and Yahr stage (HY) (15, 16), Unified PD Rating Scale (UPDRS)-I (15), UPDRS-II (15), depression (16), and recognition (16). However, some other studies believed that sarcopenia had no correlations with disease duration (14), HY (14), UPDRS-I (14), UPDRS-II (14), depression (15, 17), and cognition (14, 15). In addition, some studies found that falls incidence (15, 16) and female proportion (20) in PD with sarcopenia were higher than those in PD without sarcopenia. However, some studies reported that there were no differences in falls incidence (20) and female proportion (14, 15) between PD patients with and without sarcopenia.

Therefore, the main objective of the current study was acted in accordance with PICOS through systematical review and meta-analysis method: participants (P): patients with a diagnosis of PD from medical institutions or population; intervention (I): PD patients with sarcopenia; control (C): PD patients without sarcopenia; outcome (O): the prevalence of sarcopenia in PD patients, and the differences in clinical features between PD patients with and without sarcopenia; and study design (S): cohort study, case–control study, or cross-sectional study.

## Methods

### Search Strategy and Selection Criteria

This systematic review was designed and reported within the Preferred Reporting Items for Systematic Reviews and Meta-Analyses (PRISMA) framework. A systematic search of Medline (PubMed), EMBASE, and Cochrane database was performed from the start of the database to 1st May 2020 and was conducted in line with the Meta-analysis of Observational Studies in Epidemiology (MOOSE) criteria. The search strategy included MeSH terms and keyword variations, to identify all studies investigating sarcopenia in patients with PD. The following strategy was used in the PubMed database: ((sarcopenia [Title/Abstract] OR muscular atrophy [Title/Abstract] OR muscle Loss [Title/Abstract] OR decreased muscle [Title/Abstract] OR muscle weakness [Title/Abstract]) AND (Parkinson's disease [Title/Abstract] OR Parkinson Disease [Title/Abstract] OR parkinsonism [Title/Abstract] OR parkinsonian [Title/Abstract])). The search was limited to English-language publications. The inclusion criteria were as follows: (a) type of population: enrolled subjects with a diagnosis of PD, (b) definition of sarcopenia: measured sarcopenia assessed using a formal operationalized measure, and (c) type of study: prospective cohort study, case–control study, or cross-sectional study. The exclusion criteria were as follows: (a) type of population: populations or patients with parkinsonian symptoms, but without formal PD diagnoses, (b) definition of sarcopenia: studies that defined sarcopenia according to a non-objective or non-standardized manner, and (c) type of study: reviews, editorials, case studies, and conference abstracts.

Two reviewers (Feng, Cai) evaluated each abstract for inclusion according to these criteria, and of selected abstracts, full publications were then obtained and reviewed in detail by the same two independent reviewers. Any differences were resolved by discussion and referred to a third researcher (Jiang) when required.

### Data Extraction and Quality Assessment

After inclusion, the following information was extracted from each study to a data extraction form: study date, sample size, demographic characteristics of subjects, number of people in the sample with PD, the sarcopenia measure used, and findings regarding sarcopenia. Study authors were contacted when required to provide further information.

### Risk of Bias Assessment

Articles included in the study were assessed for risk of bias using two domains of the Quality in Prognosis Studies tool (25) that are relevant to observational studies (1. study participation and 2. outcome measurement). Appraisal of each domain provided a subjective assessment of risk of bias (ranked as low, moderate, or high). A summary of the areas considered in the assessment of each domain was included in Table 1.

**Table 1:** Characteristics of studies included in the review.

**References**	**Country**	**Sampling frame**	**N (PD/total)**	**PD participant's characteristics**	**Disease duration Years^**a**^**	**Levodopa equivalent daily dosage^**a**^**	**Mean age of PD group (SD)**	**% male of PD group**	**Risk of bias^**b**^**
**Cross-sectional study**									
Krenovsky et al. (4)	Germany	PD (C) NC (P)	53/104	HY	5.08 (1.37)	–	70 (10.1)	54.7	L/L
Ozer et al. (20)	Turkey	–	70/155	ADL, IADL	6.0 (5.1)	400 (230)	68.3 (5.9)	58.5	H/L
Peball et al. (15)	Austria	PD (C) NC (P)	104/434	HY, I–IV MDS-UPDRS, frailty, Cogn Intact, fatigue, apathy, hallucination, ADL, PDQ8 SL	12.0 (7.9)	842.6 (537.6)	73.8 (5.2)	61.5	M/M
Lee et al. (14)	Taiwan	–	52/71	I–III MDS-UPDRS, Cogn Intact, ADL	2.2 (2.2)	505.14 (376.43)	61.7 (10.6)	40.3	H/H
Yazar et al. (3)	Turkey	–	166/415	MDS-UPDRS			F 71.57 (5.2)	50	M/L
							M 72.76 (4.42)		
Tan et al. (21)	Malaysia	PD (C) NC (P)	93/171	HY, I–IV MDS-UPDRS, frailty	8.5 (5.6)	598.2 (394.2)	66.0 (8.5)	54.8	L/L
Lima et al. (16)	Brazil	C	218/218	HY, PDQ, dementia, ADL, Depr	7 (4–13)	1,000 (600–1,400)	67.9	57.3	L/M
Vetrano et al. (22)	Sweden and Italy	C	210/210	Cogn Intact, ADL, I–IV MDS-UPDRS	F 4.0 (1.5–6.7)		73.3 (7.4)	61.9	M/L
					M 3.2 (1.1–7.0)				
Barichella et al. (19)	Italy	C	235/364	–	–	–	–	–	L/L
**Prospective cohort study**									
Drey et al. (17)	Germany	P	255/255	I–IV MDS-UPDRS, RBD, Depr, hyposmia	–	–	64.9 (5.9)	59	L/L

*PD, Parkinson's disease; NC, normal control; P, population; C, clinical; SD, standard deviation; HY, Hoehn and Yahr Scale; Cogn Intact, cognitively intact; ADL, Activities of Daily Living; IADL, Instrumental Activity of Daily Living; Depr, depression; RBD, REM sleep behavior disorder; F, female; M, male; L, low; H, high; M, moderate; UPDRS, Unified PD Rating Scale*.

a *Mean ± standard deviation for normally distributed data and median (25th−75th) for not normally distributed data*.

b *Risk of bias assessed using two domains of the Quality in Prognosis Studies (QUIPS) tool that are relevant to observational studies: risk of study participation bias/risk of outcome measurement bias*.

### Data Synthesis

Data analysis was performed using the Stata version 15.0 and the Review Manager 5.3 software. Heterogeneity between estimates was assessed using the *I*^2^ statistic. An *I*^2^ value above 75% indicated high heterogeneity. Meta-analysis was undertaken using a random-effects model (to account for heterogeneity) (26). A pooled prevalence figure was calculated with 95% confidence interval (CI).

Potential influences on the prevalence estimates of sarcopenia were investigated using subgroup analyses and meta-regression. We then assessed the influence on the prevalence estimates of sarcopenia by the following variables that were identified *a priori* as potential sources on the prevalence estimates of sarcopenia: (1) sarcopenia criteria, (2) geographical location, (3) disease duration, (4) risk of bias, and (5) cognition. We classified studies as being either at low risk of bias (low risk of both participants and outcome measurement bias) or at moderate-to-high risk of bias (moderate or high risk of either participants or outcome measurement bias). We compared the European studies with the rest of the studies and the Asian studies with the rest of the studies. A comparison between studies using EWGSOP and Non-EWGSOP was performed since sarcopenia criteria were varied among different studies. Furthermore, a comparison between the studies with PD patients with average disease duration of more than 5 years and the rest of the studies and studies with PD patients with average disease duration of <5 years and the rest of the studies was conducted since disease durations of PD patients were varied among different studies. A comparison between the studies with PD patients with Mini-Mental State Examination (MMSE) score more than or equal to 24 scores and the rest of the studies was conducted since cognition levels of participants were varied among studies. We ran five meta-regression models including these covariates (including sarcopenia criteria, geographical location, risk bias, disease duration, and cognition) separately using Stata version 15.0. If a sufficient number of studies were identified, evaluation for publication bias will be performed using Begg's funnel plot.

## Results

### Study Selection and Characteristics

**Figure 1:**
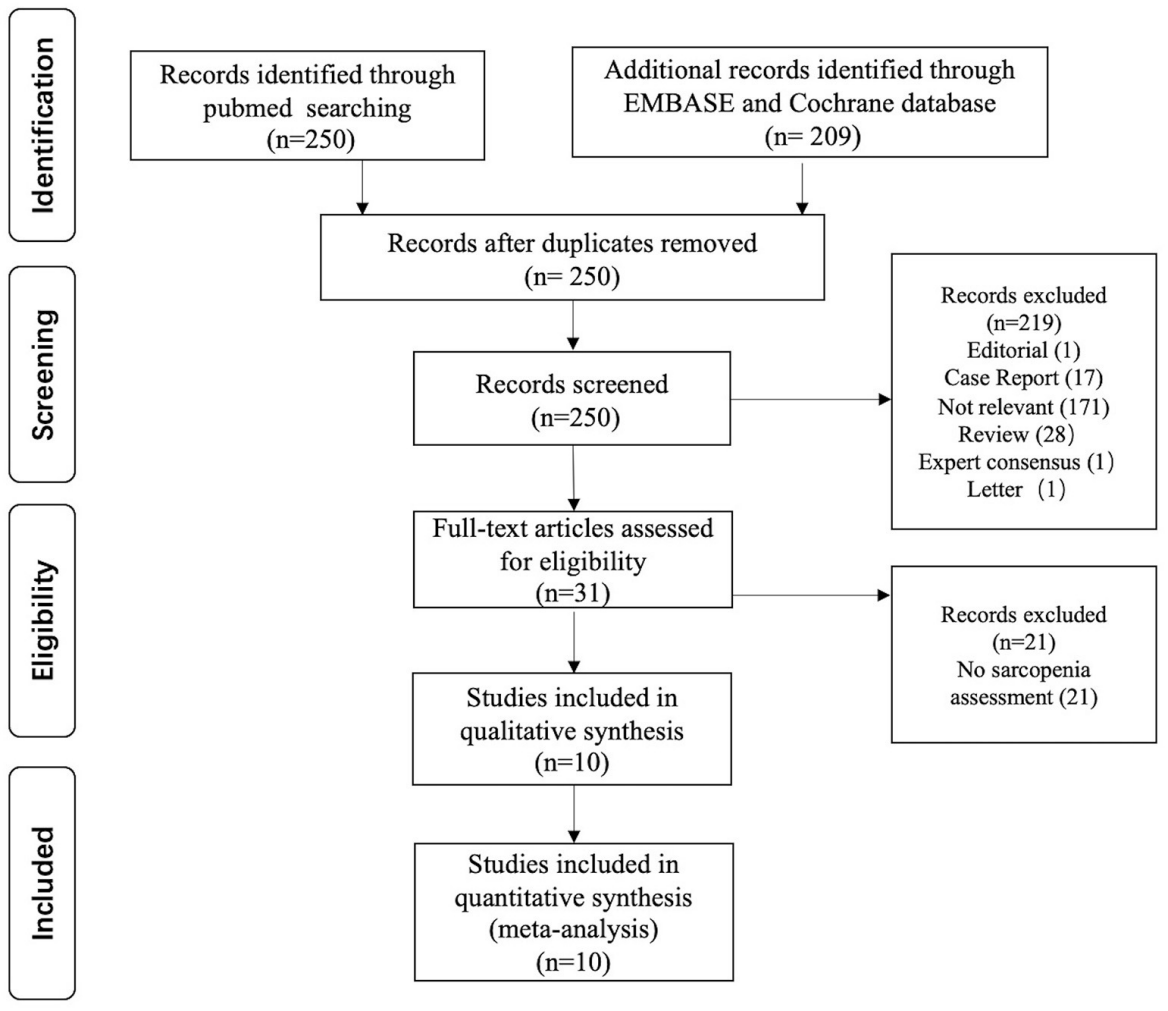
PRISMA flowchart of included studies.

A total of 459 potentially eligible articles were identified using our search strategy (250 articles from PubMed and 209 articles from EMBASE and Cochrane database). After the exclusion of 209 duplicated articles, 250 articles underwent title and abstract review. Two hundred nineteen articles were excluded at this stage since they were case reports, editorials, review articles, expert consensus, or not relevant studies. After the full-length article reviewing, 21 from 31 articles were excluded as they did not report sarcopenia assessment. Finally, nine cross-sectional studies and one prospective cohort study were included in the final analysis with a total of 2,397 participants. The literature review process is shown in Figure 1. The characteristics and quality appraisal of the included studies are presented in Table 1. The description of sarcopenia according to different criteria of included studies is presented in Table 2.

**Table 2:** Sarcopenia according to different criteria of studies in the review.

**Study**	**Sarcopenia criteria**	**Sarcopenia definition (GS: m/s; HS: kg; SMMI/ALMI/ASMMI: kg/m^**2**^)**	**Sarcopenia prevalence (%)**	**Sarcopenia measures**
Krenovsky et al. (4)	EWGSOP 1st	4-m GS <0.8; or HS male <30 female <20; and SMMI male <8.87 female <6.42	7.5	HS, GS, SMMI (BIA), Z-score sarcopenia
Ozer et al. (20)	EWGSOP 1st	4-m GS <0.8; or HS male <32 female <22; and SMMI male <10.76 female <6.76	31.4	SMMI, FMI, FFMI (BIA)
Peball et al. (15)	SARC-F	SARC-F: ≥4	58	None
Lee et al. (14)	ASMMI	ASMMI male <6.76 female <5.28	40.4	FM, fat percentage, ASMMI (DEXA)
Yazar et al. (3)	EWGSOP 1st	4-m GS <0.8; or HS male <30 female <20; and SMMI male <8.87 female <6.42	26.5	HS, GS, SMM (BIA)
Tan et al. (21)	AWGS	4-m GS <0.8; or HS male <30 female <20; and SMMI male <8.87 female <6.42	17.2	HS, GS, FM, FMI, fat percentage, SMMI (DEXA)
Lima et al. (16)	Pro-Sar	FTSTS >15 s; or HS male <27 female <16; and SARC-F: ≥4	47.2	HS, GS, FTSTS
Vetrano et al. (22)	EWGSOP 1st	4-m GS <0.8; or HS male <26 female <16; and ALMI male ≤7.23 female ≤5.67	24.3	HS, GS, ALM, ALMI, ALM_BMI_ (DEXA)
Barichella et al. (19)	EWGSOP 1st	4-m GS <0.8; or HS male <30 female <20; and SMMI male <8.87 female <6.42	6	HS, GS, SMM, SMMI (BIA)
Drey et al. (17)	ESS	20-m GS or HS and SMMI that were in the lower half of the scales	38	HS, GS, SMMI (BIA)

*EWGSOP, the European Working Group on Sarcopenia in Older People criteria; SARC-F, a tool for screening sarcopenia risk, including five items (strength, walking ability, rise from a chair, climb stairs, and falls); ASMMI, the appendicular skeletal muscle mass index; AWGS, Asian Working Group on Sarcopenia algorithm criteria; Pro-Sar, probable sarcopenia; ESS, early stage sarcopenia; FTSTS, Five Times Sit-to-Stand; HS, handgrip strength; GS, gait speed; SMMI, the skeletal muscle mass index; ALMI, appendicular lean bone-free mass index; FMI, fat mass index; FFMI, fat-free mass index; FM, fat mass; SMM, the skeletal muscle mass; ALM, appendicular lean bone-free mass; ALM_BMI_, AMI by body mass index (BMI); BIA, bioelectrical impedance analysis; DEXA, dual-energy X-ray absorptiometry; 1st, 1st version*.

### Risk of Bias

A summary of the risk of bias of the included articles is provided in Table 1. Four studies (40%) were considered to be at low risk of bias for both study participants and outcome measurement, and one study (10%) (14) was considered to be at high risk of bias for both domains. Two studies (15, 16) were considered to be at moderate risk for outcome measurement bias. Seven studies (3, 4, 17, 19–22) were considered to be at low risk for outcome measurement bias that used clearly defined diagnostic criteria, reliable and validated instruments, and a similar method and setting of outcome measurement for all participants.

### Population

One prospective cohort study sampled patients (255 PD patients) from the general population (17), and six cross-sectional studies sampled patients (913 PD patients) from the clinical cohort (hospitals, outpatients, or nursing facilities) (4, 15, 16, 19, 21, 22), whereas three cross-sectional studies did not declare the type of sampled patients (288 PD patients) (3, 14, 20) in Table 1.

### Geographical Variation

In Europe, the pooled prevalence of sarcopenia in PD patients was 0.19 (0.04–0.34). In Asia, the pooled prevalence of sarcopenia in PD patients was 0.20 (0.19–0.36) (Table 3).

**Table 3:** Results of subgroup analyses and three separate meta-regression analyses based on sarcopenia criteria, location, disease duration, sex, risk of bias, and cognition.

		**Subgroup analyses**			**Meta-regression**	
		**Number of estimates**	**Pool estimate (95% CIs)**	***I*^**2**^, %**	**Mean difference (95% CIs)**	***P***
All estimates		10	0.29 (0.18–0.40)			
Sarcopenia criteria		10			0.2080357 (0.01682, 0.3992513)	0.036^*^
	EWGSOP		0.19 (0.08–0.29)			
	Non-EWGSOP		0.40 (0.27–0.52)			
Location	Asian vs. rest	10			−0.0097027 (−0.2715711, 0.2521658)	0.934
	Asian		0.20 (0.19–0.36)	71.4		
	Rest		0.30 (0.13–0.46)	98.0		
	Europe vs. rest	10			−0.1737253 (−0.3917267, 0.044276)	0.103
	Europe		0.19 (0.04–0.34)	97.1		
	Rest		0.36 (0.24–0.48)	91.5		
Disease duration	≥5 years vs. rest	10			0.0509269 (−0.2009738, 0.3028277)	0.635
	≥5 years		0.32 (0.13–0.50)	96.1%		
	Rest		0.27 (0.12–0.41)	96.8		
	<5 years vs. rest	10			0.0334573 (−0.2884542, 0.3553088)	0.817
	<5 years		0.31 (0.15–0.47)	78.8		
	Rest		0.29 (0.15–0.42)	97.3		
Risk of bias^#^		10			−0.2026787 (−0.4042268, −0.0011306)	0.049^*^
	Low risk		0.17 (0.02–0.33)	96.7		
	Moderate/high risk		0.37 (0.27–0.48)	90.3		
Cognition		10			−0.1182179 (−0.378817, 0.1423811)	0.326
	MMSE ≥ 24		0.21 (0.04–0.38)	95.5		
	Rest of studies		0.33 (0.20–0.45)	94.9		

# *Low risk of bias: low risk on both participation bias and outcome measurement bias domains of the Quality in Prognosis Studies (QUIPS) tool; moderate/high risk of bias: moderate or high risk of bias on either participation bias or outcome measurement bias domains of the QUIPS tool. CI, confidence interval; MMSE, Mini-Mental State Examination*.

* *P < 0.05*.

### Prevalence of Sarcopenia in PD

Ten studies that provided the prevalence estimates of sarcopenia were included in the meta-analysis. Participants were not recruited from the same sampling frame, which could not lead to an overlap of study populations.

**Figure 2:**
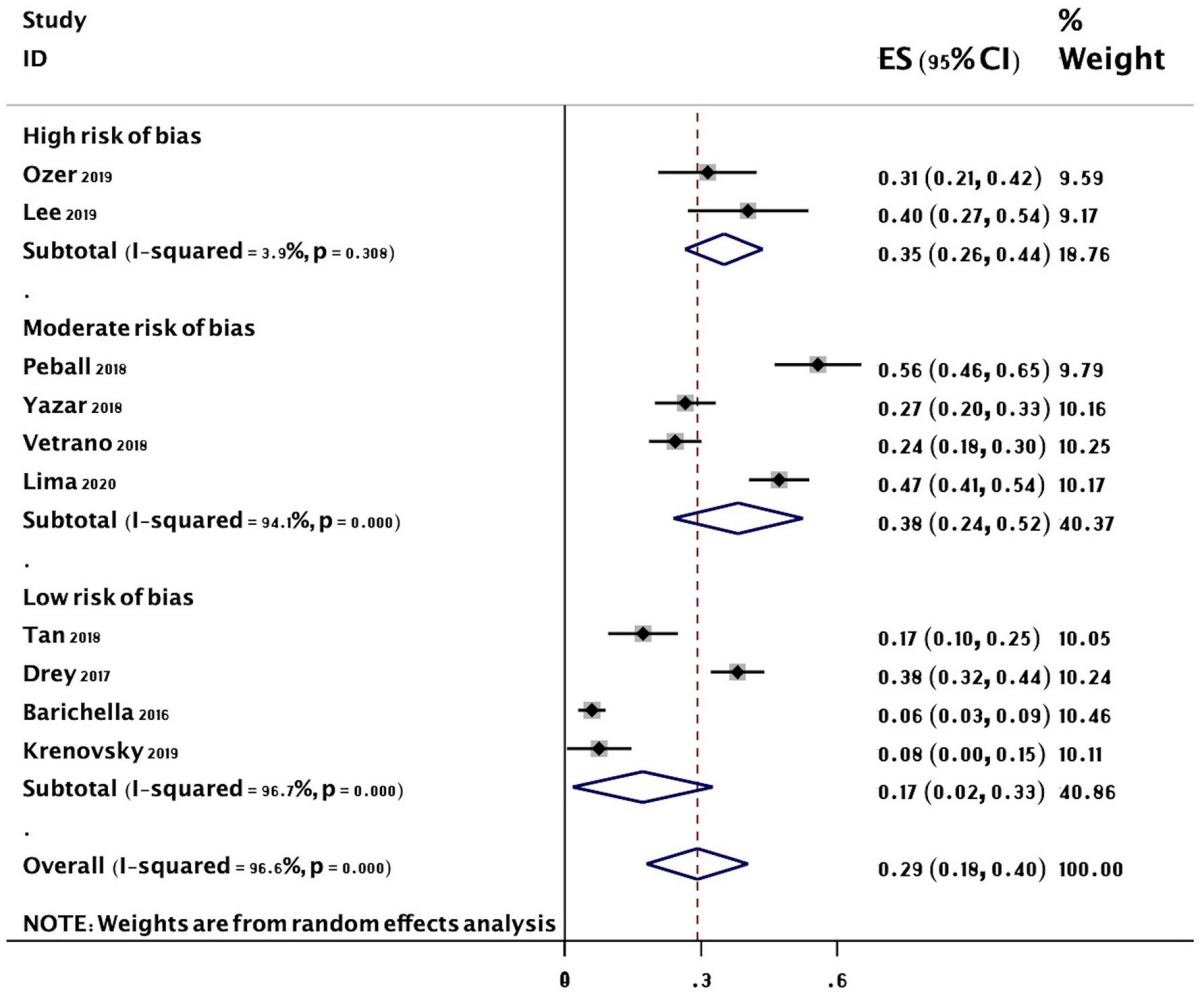
Forest plot of the prevalence (%) of sarcopenia in subjects with PD. Subgroup by risk of bias. Random-effects analysis. High risk studies are those at high risk of bias on either domain of the Quality in Prognosis Studies (QUIPS) tool. Moderate risk studies are those at either moderate risk of bias on both domains or moderate in one and low in the other. Low risk studies are those at low risk of bias on both domains of the QUIPS tool. PD, Parkinson's disease.

**Figure 3:**
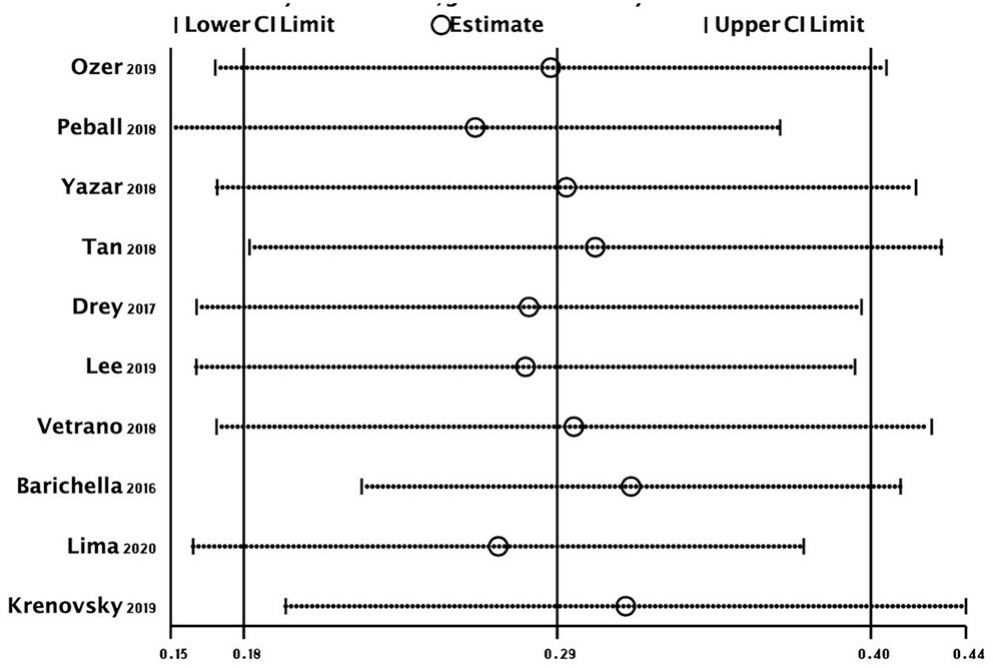
Meta-analysis estimates, given name of the study is omitted.

The overall random-effects pooled prevalence of sarcopenia was 0.29 (95% CI: 0.18–0.40) with a high level of heterogeneity (*I*^2^ = 96.6%) (Figure 2). When only studies at low risk of bias (on both domains of the Quality in Prognosis Studies tool) were selected, the pooled prevalence decreased to 0.17 (95% CI: 0.02–0.33), with still high heterogeneity (*I*^2^ = 96.7%). Similar results were obtained from all sensitivity analyses (Figure 3).

The results of five meta-regression analyses of pooled estimates of subgroups based on sarcopenia criteria, geographical location, disease duration, risk of bias, and cognition are included in Table 3. There was little evidence of an effect of location (*P* = 0.934), disease duration (*P* = 0.635), and cognition (*P* = 0.326) on the prevalence of sarcopenia. However, there was an apparent higher effect of sarcopenia criteria (EWGSOP 19 vs. Non-EWGSOP 40%, *P* = 0.036) and risk of bias (low risk 17 vs. moderate/high risk 37%, *P* = 0.049) on the prevalence of sarcopenia (Table 3).

### Comparison of Falls Incidence and Sex Between PD Patients With and Without Sarcopenia

**Figure 4:**
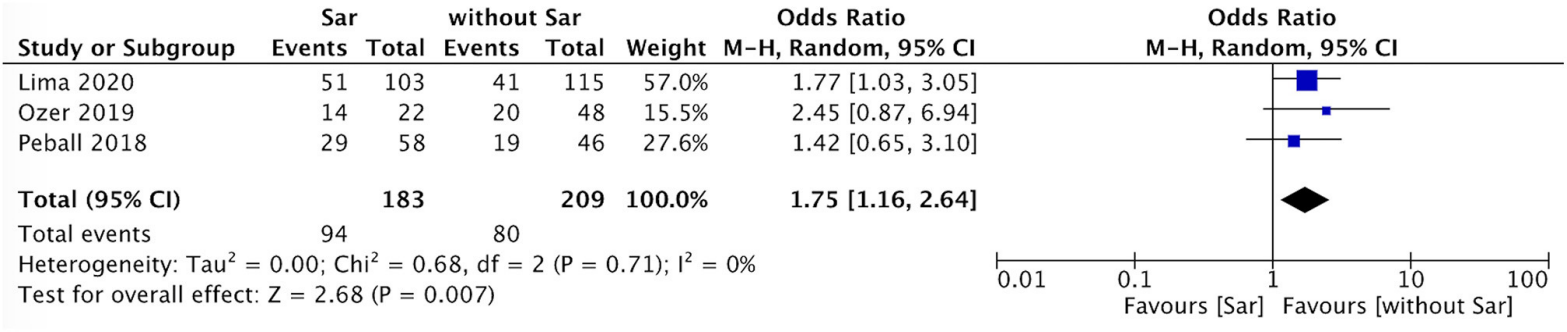
PD with sarcopenia vs. PD without sarcopenia: % fall. PD, Parkinson's disease; Sar, sarcopenia.

**Figure 5:**
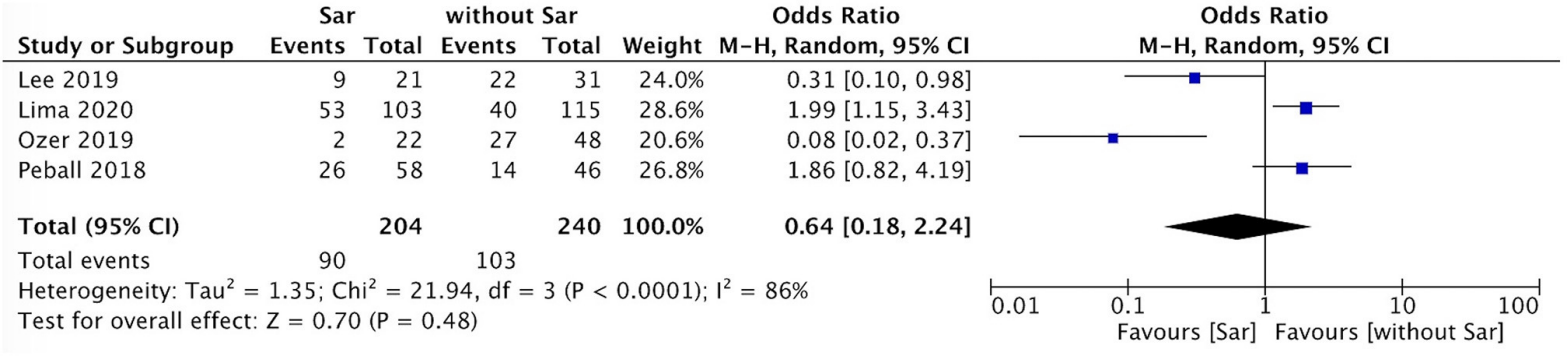
PD with sarcopenia vs. PD without sarcopenia: % female. PD, Parkinson's disease; Sar, sarcopenia.

**Figure 6:**
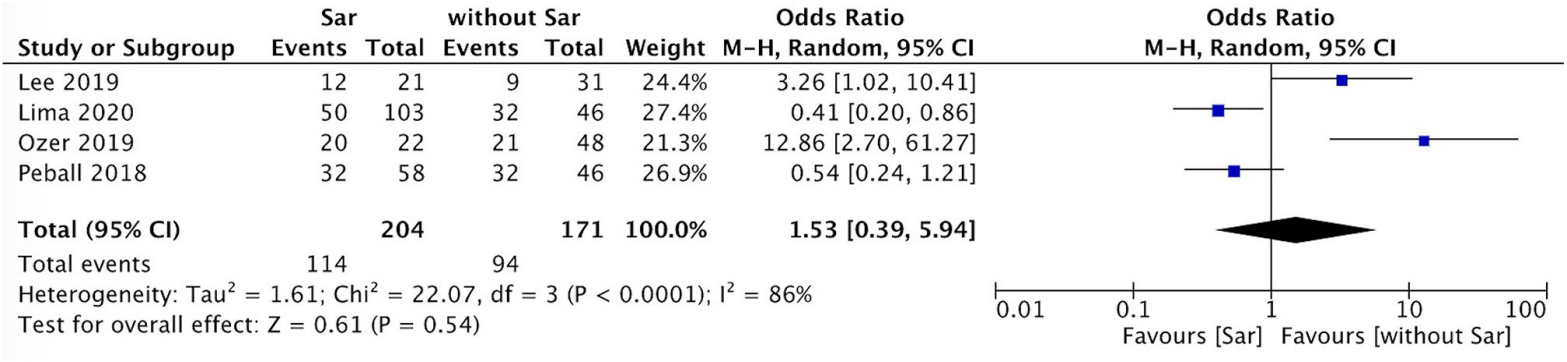
PD with sarcopenia vs. PD without sarcopenia: % male. PD, Parkinson's disease; Sar, sarcopenia.

The incidence of falls in PD patients with sarcopenia was higher than that in PD patients without sarcopenia (*P* = 0.007, *I*^2^ = 0) (Figure 4). There was no sex ratio difference between PD patients with sarcopenia and without sarcopenia (female *P* = 0.48, *I*^2^ = 86%; male *P* = 0.54, *I*^2^ = 86%) (Figures 5, 6).

### Evaluation for Publication Bias

**Figure 7:**
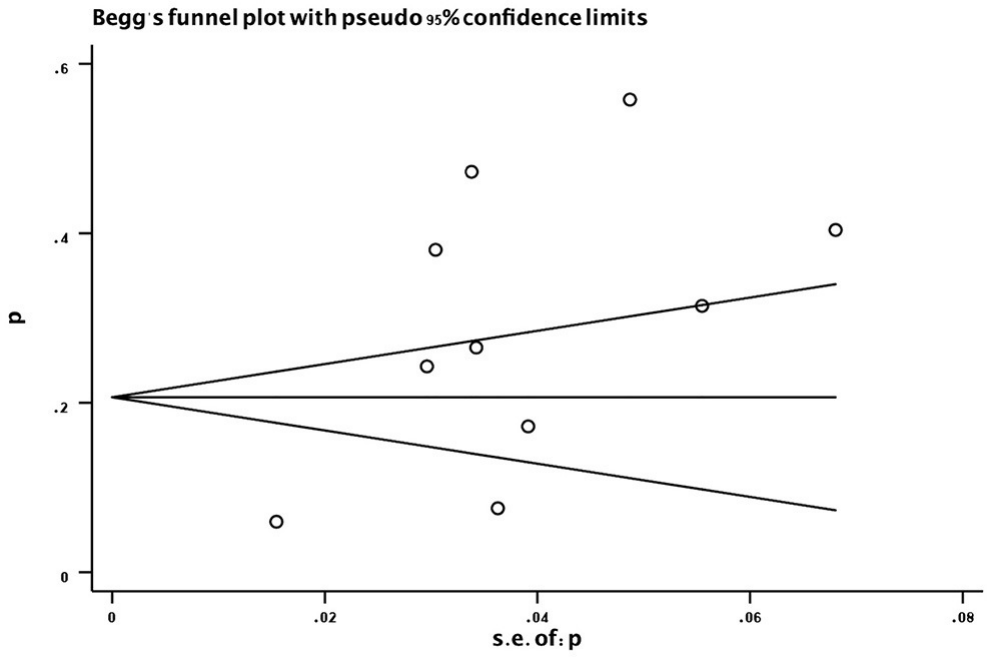
Funnel plot of the prevalence (%) of sarcopenia in subjects with PD. p, prevalence; s.e., standard error; PD, Parkinson's disease.

Evaluation for publication bias was used by Begg's funnel plot (Figure 7). No significant publication bias was found (*P* = 0.371).

## Discussion

To my best knowledge, this is the first systematic review of sarcopenia in PD. Despite its clinical importance, sarcopenia in PD patients has not been much explored in clinical practice. Only 10 papers in accordance with the inclusion and exclusion criteria were enrolled in the meta-analysis.

Our study mainly found that the pooled prevalence of sarcopenia was 29% in PD, which was higher than the healthy older control group (3, 20, 21). There are several probable mechanisms to explain the high coexistence between the two conditions.

Firstly, sarcopenia and PD may share common neuroinflammation pathways. Elevated levels of circulating inflammatory mediators were detected in the early stages of both PD patients and patients with sarcopenia (27). Interleukin-6 has been reported to be associated with muscle loss and poor physical performance in patients with PD (27). Secondly, the changes in brain structure and network were considered to play a critical role in the pathophysiology of PD patients who have sarcopenia. Wu et al. found that the gray matter volume reductions in specific regions, such as the uncus and superior temporal gyrus, were significantly associated with an increased fat percentage of the thigh and the decreased superior temporal gyrus volume was associated with an increased fat percentage of the thigh in PD patients (28). An increased fat percentage of the thigh means fatty infiltration and represents core muscle loss in PD patients. Decreased volume of the default mode network causes the insufficient activity of the task-related network when performing a task, consequently resulting in poor motor function (28). Fractional anisotropy values were also decreased in the regions of the right parahippocampal gyrus and left occipital and right temporal white matter (WM) in PD patients with sarcopenia compared with PD patients without sarcopenia by using the Diffusion Tensor Imaging technique (14). Fractional anisotropy values reduction in the left cingulum and right anterior thalamic radiation in PD patients with sarcopenia exhibited the strongest correlations with decreased muscle mass (14), which represented WM alterations in the executive functional network in PD patients with sarcopenia (14). Moreover, decreased ASMMI was associated with reduced fractional anisotropy in the fronto-striato-thalamic circuits in PD patients with sarcopenia (14).

Thirdly, besides affecting the central nervous system, the decrease in the numbers of motoneurons, i.e., mild motor neuron degeneration, might be another mechanism for neurogenic sarcopenia in PD since a low number of motor units were observed only in PD patients compared with controls (29). Neurogenic sarcopenia was considered as a subgroup of sarcopenia with a reduced number of motor units, which may indicate that sarcopenia and PD may have overlapping pathophysiological mechanisms for decreased numbers of motor neuron (4). Fourthly, sarcopenia may be influenced by the hormonal alterations in PD. Androgen plays an important role in the maintenance of muscle mass. Low plasma testosterone levels can cause or accelerate muscle- and age-related diseases, such as sarcopenia (30). Nevertheless, no study has been conducted to explore the relationship between testosterone and sarcopenia in PD. Future study is needed to elucidate it. Moreover, gastrointestinal infections, such as *Helicobacter pylori* and small intestinal bacterial overgrowth in PD (31, 32), have potentially relevant effects on body weight and neurogastrointestinal hormones. However, leptin, ghrelin and GLP-1 may not play a major role in altered body composition in PD (21).

Lastly, several PD clinical features may affect the body composition and physical performance of people with PD. PD patients have lower levels of physical activity (in terms of amount and intensity) than healthy older adults (3, 17). Malnutrition affects up to 24% of PD patients. Anorexia, nausea, constipation or delayed digestion, depression, and some pharmacologic treatment concur to reduce energy intake (22). However, the current meta-analysis cannot perform subgroup analysis to exclude the effect of anti-parkinsonism medication.

Fall was common in PD and associated with disease duration, freezing of gait, postural instability, non-motor symptoms, high levodopa equivalent daily dosage (LEDD), and greater number of medications (16, 20). The frequency of fall significantly worsens the outcome of PD (33). Several studies have shown that reduced mobility, poor balance, and reduced leg muscle strength increased fall risk (34, 35). These signs are clinical manifestations of sarcopenia. Our current meta-analysis also found that the frequency of falls in PD patients with sarcopenia was higher than that in PD patients without sarcopenia. Furthermore, PD patients with probable sarcopenia and falls have been reported to have higher HY staging and lower Schwab and England Activities of Daily Living scores (16). Therefore, sarcopenia and fall in PD may interact with each other and establish a vicious circle.

Some studies on corticospinal activity have been performed in PD patients and healthy controls during gait. One study found corticospinal control of human locomotion as a new determinant of age-related sarcopenia through comparison of corticomuscular coherence (CMC) between sarcopenic and non-sarcopenic older adults during gait, which may hint at a novel possible mechanism derived from corticospinal control of locomotion in age-related sarcopenia (36). Another study found that older healthy controls and PD participants had significantly decreased CMC and electromyography (EMG) power at low beta frequencies (13–21 Hz) compared with young healthy controls, whereas there was no difference between the older healthy and PD groups (37). Additionally, one study found that PD participants had significantly decreased beta frequencies (16–31 Hz) for tibialis anterior muscle compared with older healthy controls, whereas there was no difference in the magnitude of CMC between older and younger healthy controls (38). This might hint toward one of the potential features underpinning gait speed changes and risk of falling in PD patients with sarcopenia. However, the limitation of the latter two studies did not screen sarcopenia in PD patients. In the future, more studies should be worked on it.

Two studies found no sex difference in PD patients with or without sarcopenia (14, 15). Wu et al. revealed that female sex was associated with core muscle loss in PD patients (28). One study found that higher fat infiltrations were detected in the psoas and thigh muscles in female PD patients compared with female healthy controls (17). Another study found that female sex was independently associated with sarcopenia (16). The current meta-analysis did not find a difference in both male and female sex ratios between PD with and without sarcopenia. However, we have to recognize the small sample size of these enrolled studies. Thus, it is necessary to conduct a study with a large sample size of PD patients and sarcopenia to address the role of sex.

Among enrolled studies, several studies found no difference in SMMI between PD patients and healthy controls (4, 20, 21) and among different stages of PD (20); PD patients with sarcopenia had higher UPDRS-III scores (14, 15, 17), lower fat-free mass index (FFMI) (20), lower ASMMI (14), and lower GS (15) than PD patients without sarcopenia. Moreover, few studies reported that sarcopenia was associated with osteoporosis (16), tremor dominant/non-tremor dominant (15), nursing home placement (15), and Parkinson's Disease Questionnaire (PDQ) (15, 16) in PD patients. Some studies found that sarcopenia in PD had no relationship with UPDRS-IV (15), orthostatic hypotension (15), hallucinations (15), LEDD (14, 16), REM sleep behavior disorder (RBD) (17), hyposmia (17), motor physical therapy (16), and social support (16). Therefore, due to the small number of studies, insufficient information of PD patients, and different evaluation standards and statistical methods, some factors, such as GS, HY, depression, LEDD, UPDRS-I, and UPDRS-II, could not be made meta-analysis in this review.

## Limitation

There were some limitations of the current study. First, only a small number of papers were enrolled, which also indicated the scarcity of research on this area. Second, most of the studies were cross-sectional study, and the cross-sectional design was not truly appropriate for addressing a cause–effect relationship between sarcopenia and disease-related factors. Prospective studies are required to address this issue. Third, the participants of nine studies were from the hospital and similar medical institutions. Therefore, the prevalence rates might not be generalizable to the overall PD population, and we could not conclude the definitive prevalence of sarcopenia in patients with PD. Fourth, several studies excluded patients with poor cognitive function and using a wheelchair. As a result, the prevalence of sarcopenia might have been underestimated in these studies. Fifth, as the effect of no worldwide consensus sarcopenia criteria, geographical location, risk bias, and population, high heterogeneity was found among the included studies. Sixth, language selection (only articles in the English language were selected) was also the limitation of this meta-analysis.

## Conclusion

Sarcopenia seems to be common in patients with PD. However, the current evidence is not enough to conclude the definitive prevalence of sarcopenia in patients with PD. The progressive loss of function associated with sarcopenia may eventually boost the neurodegenerative process in PD. It is necessary to optimize the assessment of sarcopenia and still needed to improve the sarcopenia screening procedures in PD patients. The assessment, prevention, and, hopefully in the future, treatment of sarcopenia may help to improve the prognosis of patients with PD.

## Data Availability Statement

The raw data supporting the conclusions of this article will be made available by the authors, without undue reservation.

## Author Contributions

YC, FF, QW, and ZJ designed the study and did the literature review. YC did the statistical analysis, edited tables and pictures, and wrote the primary manuscript. RO contributed to reviewing the grammar. ZJ and QW helped to promote the methodology. HS reviewed the final manuscript. All authors contributed to the article and approved the submitted version.

## Conflict of Interest

The authors declare that the research was conducted in the absence of any commercial or financial relationships that could be construed as a potential conflict of interest.
