# Supplementary material for: Sarcopenia in Patients With Parkinson's Disease: A Systematic Review and Meta-Analysis
Source: Front Neurol. 2021 Mar 5;12:598035. doi: 10.3389/fneur.2021.598035 (PMC7973225; doi:10.3389/fneur.2021.598035)
Supplement: Supplementary file 1 [file Table_1.DOCX]

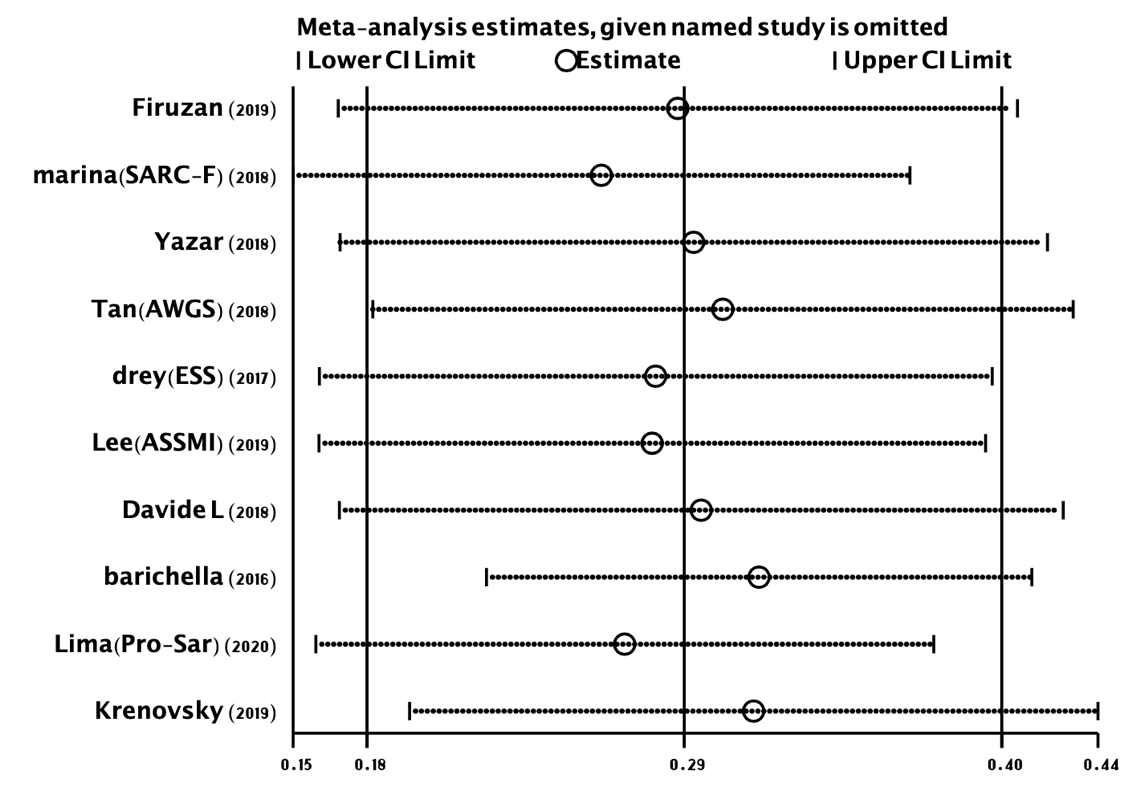


Appendix 1. Meta-analysis estimates, given named study is omitted


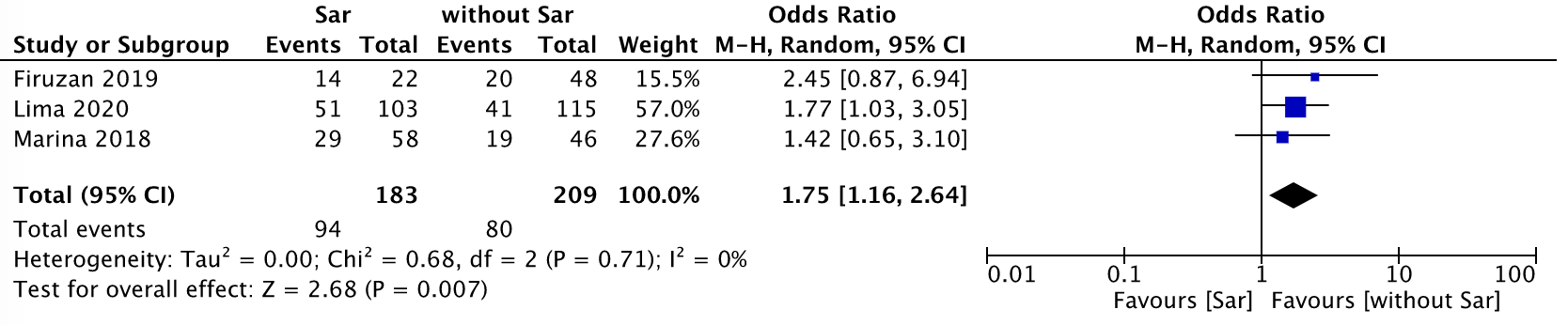


Appendix 2. PD with Sarcopenia vs. PD without Sarcopenia: %Fall


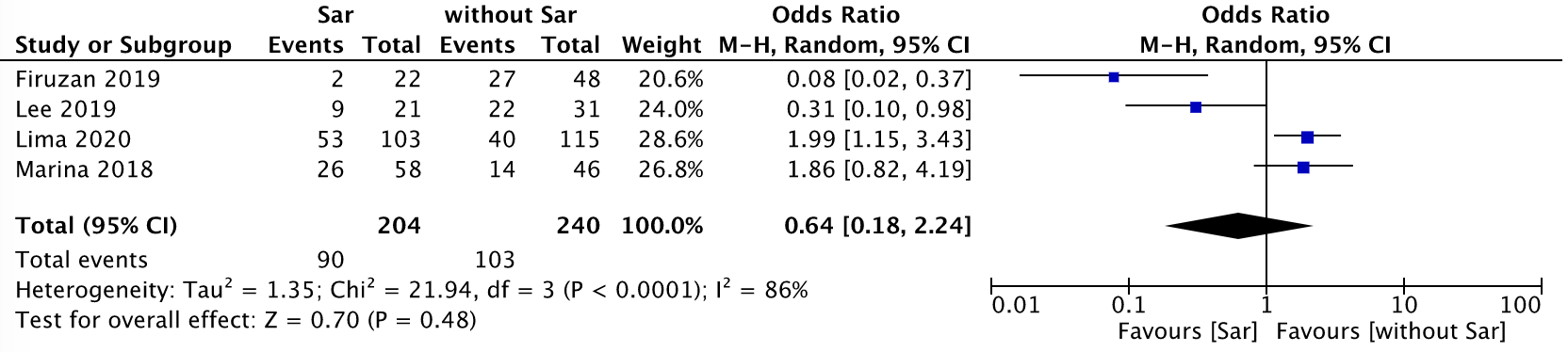


Appendix 3. PD with Sarcopenia vs. PD without Sarcopenia: %Female


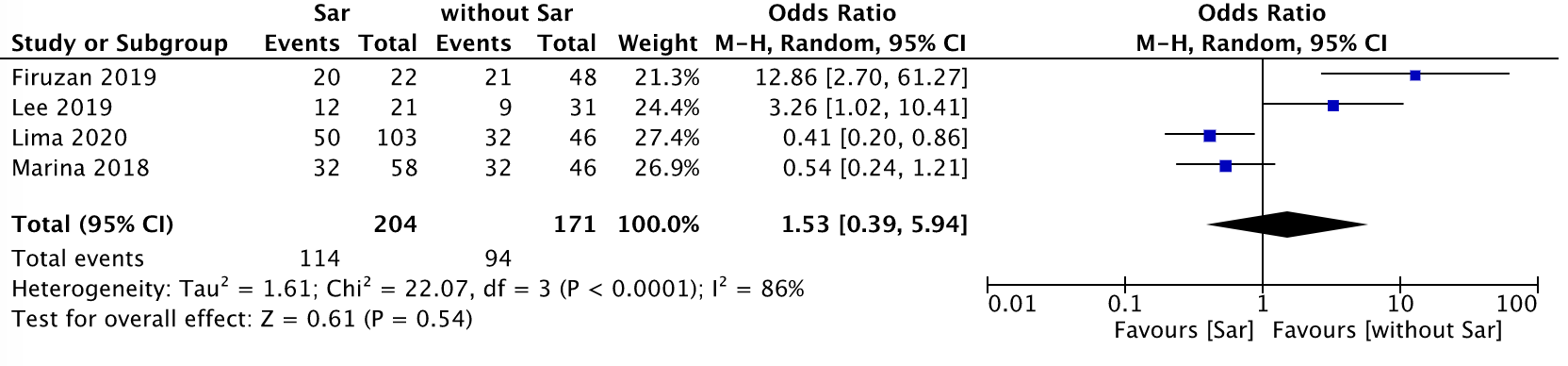


Appendix 4. PD with Sarcopenia vs. PD without Sarcopenia: %male


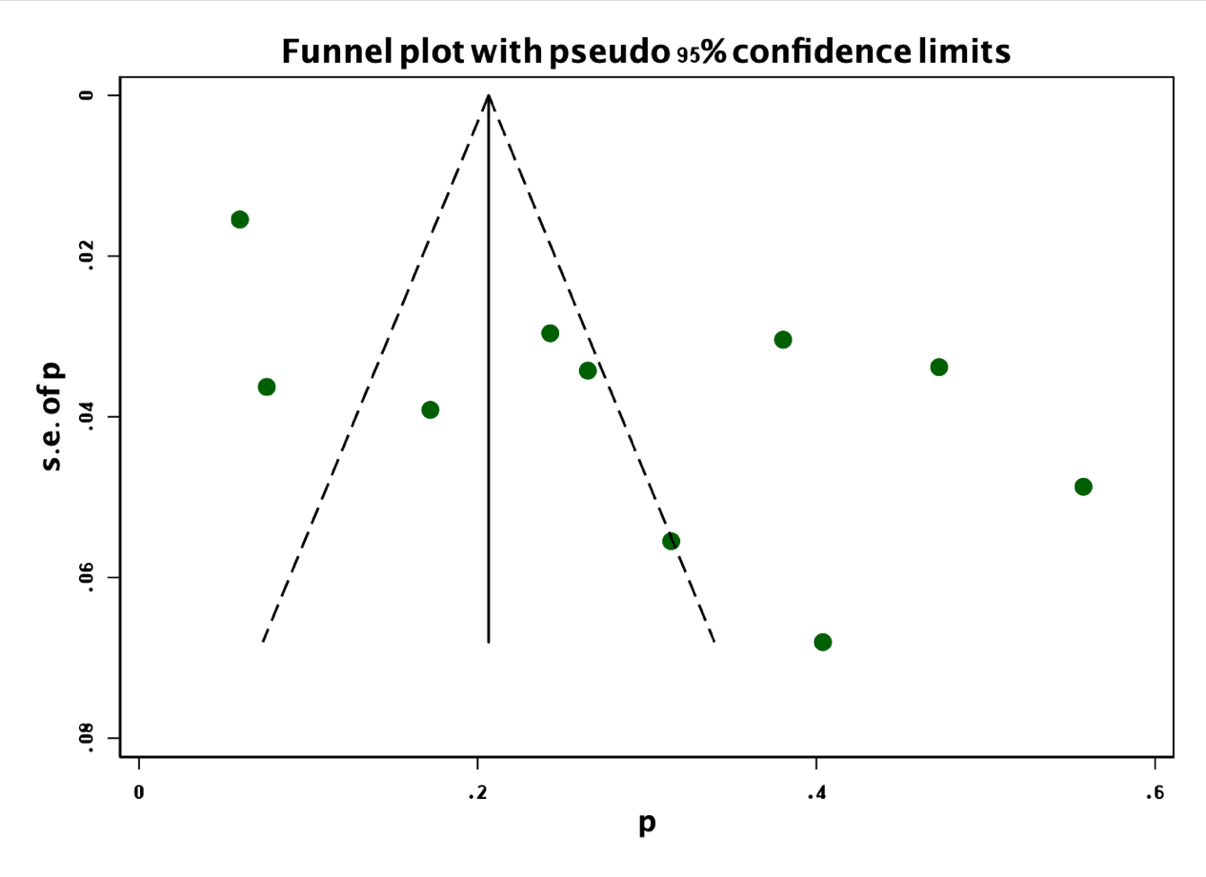
 Appendix 5. Funnel graph prevalence (%) of sarcopenia in subjects with PD
